# Contextual violence and its link to social aggression: a study of community violence in Juárez

**DOI:** 10.7717/peerj.9162

**Published:** 2020-07-02

**Authors:** Jaime Martín del Campo-Ríos, Christian Enrique Cruz-Torres

**Affiliations:** 1 Universidad Autónoma de Ciudad Juárez, Instituto de Ciencias Sociales y Administración, División Multidisciplinaria de Ciudad Universitaria, Ciudad Juárez, Chihuahua, Mexico; 2 Universidad de Guanajuato, Campus León, León, Guanajuato, Mexico

**Keywords:** Contextual victimization, Community violence, Aggression, Post-traumatic stress disorder

## Abstract

The city of Juárez, Mexico has been immersed in an atmosphere of violence and danger for more than a decade. Due to this violence, residents of Juárez may be at risk of severe contextual victimization, which occurs when individuals are indirectly affected by the physical and socio-cultural conditions of their violent communities through second-hand information (e.g., witnessing or hearing about violent acts in their everyday life). The objective of this study was to explore the effects of contextual victimization on variables related to community violence such as aggression, post-traumatic stress disorder (PTSD), and acceptance of violence. Data were collected from a sample of university students in Juárez (*n* = 298) using the Aggression Questionnaire (AQ), the Acceptance of Violence Scale (AVS), Checklist for PTSD Traits scale, and the Contextual Victimization by Community Violence scale (CVCV). Participants’ responses were analyzed in structural equation models (SEM) to uncover the latent variables behind each scale and test the hypothesized effects of CVCV on PTSD, AQ and AVS. Good validity indexes and internal consistency of all instruments were confirmed. SEM show significant positive effects of contextual violence on PTSD and PTSD on the disposition to aggression, but not on the acceptance of violence. Also, the variance explained of PTSD and AQ found in the sample of women (20% of PTSD and 23% of AQ) is almost twice than in men’s sample (9% for PTSD and 14% for AQ).

## Introduction

### Demographics of violence in Juárez, Mexico

The Mexican Drug War has caused some of the most disturbing concentrations of urban violence in the past two decades. From 2008 to 2012, the epicenter of this phenomenon was the border city of Juárez, which suffered historic levels of crime including high rates of femicide, armed assaults, car-jackings, extortions and kidnapings. The collateral results of this violence were an estimated 10,000 children who were orphaned, 250,000 residents who fled from the city, 10,000 businesses which closed, 30,000 jobs lost and 25,000 houses which were abandoned (Aziz Nassif, 2012); in other words, everyday life in Juárez became paralyzed, affecting all social strata of the city. There were more than 10,000 homicides in this period of time, which represents a quarter of all drug-related deaths in Mexico, more than the number of civilian casualties in Afghanistan over the same period, and more than double the number of U.S. troops killed in the entire Iraq war (Eisenhammer, 2014). By 2008 and 2009, homicides in Juárez were estimated to represent 27.6% of those committed in the entire country (Barraza, 2009). Current state of community violence in Juárez has significantly decreased, although it is still perceived as an unsafe city. According to the National Institute of Statistics and Geography 78.2% of its population considers it a dangerous place to live (National Institute of Statistics and Geography (INEGI), 2018). Monthly homicides data of Juarez show that the rates reached their last peak over 400 homicides per month in October 2010 (477 homicides registered), and although the numbers started to decrease since then, monthly homicide rate fell by almost half, with 235 homicides. This general trend endured until present times (National Institute of Statistics and Geography (INEGI), 2019).

### Types of violence exposure and effects

Echeburúa (2004) conceptualizes three different types of victims resulting from violence exposure “(a) Direct victims or primary sufferers. People directly affected by the aggression or the traumatic event; (b) Secondary or indirect victims. Included in this category are family members and people that are close to the direct victims: those who are traumatized by the physical and socio-cultural conditions after directly witnessing the violence; and (c) Contextual victims or affected: those who are traumatized by the physical and socio-cultural conditions of their violent communities, this category includes people who have been psychologically affected by serious events without suffering any direct losses or threats to their lives or their family” (Gurrola-Peña et al., 2018, p. 2).

Research provides evidence that the aforementioned types of violence exposure are associated with an array of neurobiological, psychological and attitudinal phenomena. For example, the consequences of child maltreatment (i.e., primary or direct victim) can go so far as altering children’s neurobiological development (Perry, 1997; Teicher & Samson, 2016). Furthermore, specific cases of physical, sexual and emotional abuse and physical and emotional neglect, exert a prepotent influence on the trajectories of brain development, and constitute a major risk factor for adult psychopathology (Teicher & Samson, 2016). Many studies have reported that child witnesses of homicides (i.e., secondary or indirect victims) can develop symptoms of post-traumatic stress disorder (PTDS) that include re-experiencing traumatic events in play and dreams, or when exposed to intrusive images and sounds associated with the events (Pynoos & Eth, 1984; Pynoos & Nader, 1988). Witnessing acts of violence affects adults too. Shahinfar, Kupersmidt & Matza (2001) report that people exposed to violent acts tend to perceive positive outcomes of being aggressive and increase reactive aggression via hostile attribution (e.g., the tendency to interpret others’ behaviors as having hostile intent) and response selection (e.g., the process of choosing the appropriate action to take in response to a given stimulus) (Calvete & Orue, 2011). On the other hand, effects associated to contextual victimization have not been thoroughly explored as to the effects of direct and indirect violence exposure.

### Objectives and hypothesis

It is widely believed that “children learn what they see”…. (or hear). This is an unfortunate truth for Juárez in particular, where inhabitants of all ages have been exposed—whether directly or indirectly—to violence in their everyday lives. It is believed that cities with higher levels of violence permeate an acceptance and tolerance of violence which appears to be strongly allowed by cultural values considering it as a valid and even natural way to manage conflicts. These cities face a significant risk for these values becoming standards (Hernández, Ramos Lira & Méndez, 2004).

Based on the theoretical and contextual background of the violence suffered in Ciudad Juárez in recent years, we propose the following research question:

Can contextual victimization (e.g., listening or witnessing violent acts; those exposed to contextual violence specifically, not exposed to direct or indirect violence) generate emotional effects that lead PTSD, higher levels of aggression and acceptance of violence?

The hypothesis of this study is that contextual victimization influence and alter the development of social information-processing mechanisms resulting in (a) an increasement in the emotional affectations of citizens evaluated for PTSD (via the *Checklist for PTSD Traits Scale* (Pineda et al., 2002)); (b) a major acceptation of violence (reflectled in higher scores on the Acceptance of Violence scale (AVS, Velicer, Huckel & Hansen, 1989)); (c) the disposition to being violent against others (predicting higher scores on the Aggression Questionnaire (AQ) (Buss & Perry, 1992); and (d) given that the violence in Ciudad Juárez presents itself in different modalities such as rape and femicide, it is hypothesized that there will be possible differences in predictive models between men and women. As a secondary objective, a big five personality scale (Ten-Item Personality Inventory; TIPI) was implemented in order to test an alternative explanation derived from individual differences to the sensibility to threatening cues (e.g., contextual violence).

## Methods and Instruments

### Participants

Initially, a non-random sampling was performed with a recruitment of participants via flyers, email invitations and snowball sampling. Three-hundred seventy-five undergraduate students from the Autonomous University of Ciudad Juárez participated in the study. Data of 77 respondents were discarded and not considered for the statistical analyses due to participant mortality, corrupted data, or inconsistent scores on some “trap” items. Thus, 298 participants were considered for the analysis; this sample was 52.3% male and 47.7% female, and had an average age of 19.28 years (SD = 0.50). Participants were pursuing diverse majors such as business administration, systems engineering, accounting, psychology and graphic design. All were residents of Ciudad Juárez who had resided in the city for an average of 17.6 years (SD = 6.01). Among the sample, 95.3% of participants reported having lived in Ciudad Juárez between 2008 and 2012.

### Procedure

The Universidad Autónoma de Ciudad Juárez granted full ethical approval to carry out the study within its facilities and with its students (Ethical Application Ref: CIEB-2019-1-021). All measures were group-administered (20–25 individuals per session) via an electronic survey system (Survey Monkey) in a university computer lab. Respondents were physically separated during the completion of the questionnaire, and all respondents were asked to put their mobile phones on Airplane Mode. General instructions were read aloud and respondents were encouraged to ask the experimenter if they had any questions during the session. Written consent was obtained for all participants. They were informed and reassured that their responses would remain confidential.

### Instruments

Five scales constituted the main instruments used in the current study.

The 34-item, 5-factor Contextual Victimization by Community Violence Scale (CVCV; Gurrola-Peña et al., 2018) was designed to identify the frequency with which young adults have been contextual victims of crimes committed in their social environment. Previous validation of the instrument has yielded a reliability estimate of Cronbach’s α = 0.94 for item totals, and 59.52% of the total variance explained by five factors. The first factor (eight items) evaluates non-witness contextual victimization (e.g., “I have heard that in places where I go to have fun, someone was shot”), explains 39.19% of the total variance, and has a Cronbach’s α of 0.90. The second factor (seven items) focuses on witness contextual victimization (e.g., “I have seen how a person has been wounded with a weapon in the places that I frequent”), explains 7.28% of the total variance and has a Cronbach’s α of 0.85. The third factor (eight items) centers on contextual victimization in the neighborhood (e.g., “I have heard that in my neighborhood people have been kidnaped”), explains 5.45% of the total variance and has a Cronbach’s α of 0.88. The fourth factor (six items) concerns contextual victimization in recreational places (e.g., “I have heard that in places where I usually go for fun people have been killed”), explains 4.64% of the total variance and has a Cronbach’s α of 0.86. The fifth factor (five items) relates to victimization at school (e.g., “I have heard that someone at my school has been shot”), explains 3.04% of the total variance and has a Cronbach’s α of 0.80.

The next instrument was the AQ (Buss & Perry, 1992), which has been widely used to assess different dimensions of aggression. It includes 29-items scored on a five-point Likert scale (1 = uncharacteristic of me, 5 = very characteristic of me) grouped into four factors: (a) anger (A; seven items), (b) hostility (H; eight items), (c) verbal aggression (VA; five items) and (d) physical aggression (PA; nine items). The version used in this study was validated with a young sample of Mexicans (Matías et al., 2013), and is based on the AQ for Spanish pre-adolescents and young adults (AQ) (Santisteban & Alvarado, 2009). Both maintain the structure of Buss & Perry (1992) original version of the AQ. Internal consistency for the Mexican version of the AQ shows Cronbach’s α = 0.92, indicating that this version of the AQ instrument is valid and reliable for use with adult Mexican populations.

The third instrument, the Checklist for PTSD Traits Scale (Pineda et al., 2002), aims to diagnose PTSD symptoms in individuals that live in violent populations. The scale consists of 24 items based on the symptoms of PTSD consistent with DSM-IV criteria (e.g., “I constantly have thoughts that remind me of the unpleasant situation and cause me a lot of anguish”), scored using a four-point Likert scale (1 = totally disagree; 4 = totally agree). Pineda et al. (2002) report that the scale has good discriminant capability, high levels of sensibility and specificity, and excellent internal consistency (Cronbach’s α = 0. 97). The scale was previously validated in Mexico with a sample of youths exposed to social violence reporting Chronbach’s α = 0.92 (Chávez-Valdez, Esparza-Del Villar & Ríos Velasco-Moreno, 2020).

The fourth instrument, the AVS (Velicer, Huckel & Hansen, 1989), is a 14-item, three factor-solution scale validated in Mexico (Hernández, Ramos Lira & Méndez, 2004) that evaluates attitudes of acceptance of force and coercion to resolve conflicts; and the tolerance of the use of violence in a variety of situations. Responses are scored using a four-point Likert scale (1 = totally disagree; 4 = totally agree). The internal reliability estimate for the global scale is Cronbach’s α = 0.83. For individual factors, reliability estimates are, α = 0.89 for the acceptance of family violence factor (five items; e.g., “It is a right of the couple to hit the other in the face if they flirt with others”) ; α = 0.71 for acceptance of violent disciplinary tactics (four items; e.g., “A child who is usually disobedient should be punished physically”); and α = 0.67 for acceptance of military violence (five items; e.g., “The government must send armed soldiers to control the violent demonstrations”).

The last instrument was the TIPI (Gosling, Rentfrow & Swann, 2003), a 10-item instrument that measures responses on five factors (“Big Five” personality traits): extraversion, agreeableness, conscientiousness, neuroticism (or emotional stability) and openness to experience factors. This short inventory contains ten pairs of two trait descriptors (e.g., extraverted/enthusiastic) that aim to establish a broad coverage of traits whilst trying to avoid redundancy. Responses are scored on a seven-point scale (1 = Strongly Disagree to 7 = Strongly Agree; 5 reverse-scoring items). Adequate test–retest reliability and levels of convergent and discriminant validity and relationships with external correlates are reported in Gosling, Rentfrow & Swann (2003). Although the authors stress that the TIPI has only two items that result in low internal consistency estimates, the main goal of the scale was not to offer high alphas or good Confirmatory Factor Analysis fits. Although the TIPI is yet to be validated with a Mexican population, it has been translated and validated in several countries, always showing adequate levels of internal consistency and validity (e.g., two German versions (Herzberg & Brähler, 2006; Muck, Hell & Gosling, 2007), Dutch (Hofmans, Kuppens & Allik, 2008), Italian (Chiorri et al., 2015), Japanese (Oshio, Shingo & Cutrone, 2012) and a Spanish translation (Romero et al., 2012) among others), which makes the TIPI a widely applicable instrument with a potential for large-scale cross-cultural studies. Moreover, the TIPI was not the central instrument in this study; the only factor considered was emotional stability, given that it could approximate whether individuals are more sensitive in general and easily affected by contextual violence.

### Data analysis

Since the instruments were developed and validated in samples other than those analyzed in this study, the internal structure and consistency of each instrument were verified before the hypothesis test analysis. All responses were subjected to exploratory factorial analysis of maximum likelihood with varimax rotation. The minimum extraction criterion was set to an eigenvalue of 1. Items with loadings lower than 0.4 on all factors were discarded from the analysis. Once the factorial structure of each scale was established the internal consistency of each factor was calculated using Cronbach’s alpha. The indicators for each factor were formed by averaging the items with loadings greater than 0.4 onto their factor. Once the factors were identified, structural equations modeling (SEM) of maximum likelihood in AMOS 22 (Arbuckle, 2013) was performed to test the hypothesis of positive effects of CVCV on PTSD, and the positive effects of the CVCV on disposition to aggression, as evaluated by the AQ. Finally, given that violence in Ciudad Juárez has victimized men and women differently, possible differences in models between men and women were explored by multigroup comparison in AMOS software (Arbuckle, 2013).

A potential confusing factor is that the perception of contextual violence may be affected by individual differences, given that more sensitive people are expected to report higher levels of perceived violence. To explore this possibility, high and low groups were formed in the measurement of emotional stability of the TIPI. The sample was divided into high and low levels of this variable, then comparing the means of both groups in contextual violence measurements. If significant differences between the two groups were identified, it could be assumed that emotional stability is a strange variable that must be statistically controlled in the models.

## Results

Table 1 shows the main psychometric and descriptive statistics of each factor identified by the exploratory factor analysis on each instrument. The analysis for the CVCV questionnaire retained 33 of the original 34 items proposed by Gurrola-Peña et al. (2018), eliminating the item “I have seen someone get stabbed in my school” for loading values less than 0.4 for all the factors. The five-factor solution obtained explained 60.92% of the variance of responses, and corresponded to the conceptual content of the original scale. The first factor was called “Non-witness contextual victimization” given that it contained items related to hearing about violent events in areas related to one’s daily life (e.g., “I have heard that someone has been shot in my neighborhood”; “I have heard that in places that I usually go someone has been shot”). The second factor was named “Contextual victimization in the neighborhood” for comprising items that relate to having heard about (e.g., “I heard that someone has been shot in my neighborhood”) or witnessed (e.g., “I have seen drug trafficking in my neighborhood”) threatening situations in areas near the home. The third factor was named “Contextual victimization in recreational areas” because it contained items related to hearing about acts of violence in recreational areas (e.g., “I have heard that in places where I go for fun people have been shot”). The fourth factor was named “Contextual victimization at school” since it contains items related to hearing (e.g., “I have heard that someone has been shot in my school”) or witnessing (e.g., “I have seen how someone has been beaten in my school”) violent acts at school. The fifth factor was called “Witness contextual victimization”, because it is comprised of items that refer to having witnessed violent acts in areas related to one’s daily life (e.g., “I have seen someone being shot in places I usually frequent”; “I have witnessed a non-weapon assault in places where I usually go for fun”). To explore possible differences in contextual violence between men and women, the averages in each indicator were compared using Student’s *t*-tests. The results only identify statistically significant, although very small, differences in non-witness contextual victimization (*t* = 2.17, df = 296, *p* = 0.030, Men *M* = 2.21, Women *M* = 1.98) and contextual victimization in school (*t* = −1.98, df = 283.34, *p* = 0.048, Men *M* = 1.27, Women *M* = 1.39). The other three forms of contextual violence show averages that can be considered equivalent between both groups (*t*’s < 1.47, *p* > 0.141).

**Table 1:** Main psychometric indicators and descriptive statistics of the factors identified in the instruments through factorial analysis (*n* = 298).

Measure	Factor	Items	Variance explained (%)	Alpha	Average	SD
CVCV scale	Non-witness contextual victimization	11	19.10	0.94	2.10	0.92
	Contextual victimization in the neighborhood	8	15.49	0.91	2.46	0.94
	Contextual victimization in recreational areas	4	9.53	0.90	2.58	1.04
	Contextual victimization at school	5	8.43	0.77	1.33	0.50
	Witness contextual victimization	4	8.35	0.82	1.29	0.59
Checklist for PTSD traits scale	Outcomes	14	29.78	0.94	1.27	0.47
	Memories	8	19.70	0.91	1.53	0.68
	Event	2	9.70	0.80	1.58	0.84
AQ	Punches	7	14.49	0.82	1.61	0.67
	Paranoia	6	11.98	0.81	2.16	0.87
	Anger	5	11.93	0.83	2.1	0.92
	Arguing	5	11.33	0.83	2.0	0.81
AVS	Acceptance of violence against children	6	28.66	0.88	1.49	0.60
	Acceptance of couple violence	3	13.66	0.72	1.04	0.21
	Acceptance of military violence	3	11.52	0.66	1.35	0.52

**Note:**

AQ, aggression questionnaire; CVCV, community victimization by community violence scale; PTSD, post-traumatic stress disorder; AVS, acceptance of violence scale.

Analysis of the PTSD checklist showed a structure of two factors plus an indicator that contained only two items, explaining together 59.19% of the variance with the 24 original items. The first factor was labeled “Outcomes” given that it comprises items centered around changes in emotional state and everyday life provoked by traumatic events (e.g., After what happened, I have many difficulties in my relationships with others). The second factor was named “Memories”, containing items related to the recurrence of memories associated with the traumatic event (e.g., “I feel bad when something reminds me of the situation”) and the strategies used to cope with them (e.g., I always avoid thinking or talking about what happened.). The third factor was named “Event” and only has two items, but was kept since both items referred to having recently experienced a violent event (e.g., “Lately I have experienced at least one situation related to death or threats against my life or to other people related to me”) that was considered traumatic (e.g., “because of this situation I have experienced a lot of anguish or excessive fear”), in addition to explaining an adequate amount of variance (see Table 1).

The analysis of the AQ identified four factors consisting of 23 of the original 29 items of the scale, which together explained 49.75% of the variance in responses. Six of the items were discarded because they presented loadings of less than 0.4 on all factors. The first factor was named “Punching”, for referring to the disposition to enter into a conflict and hit other people (e.g., If they provoke me enough, I can hit another person). The second factor was named “Paranoia”, because it refers to a general distrust of other people and the possibility of being hurt by them (e.g., “When people are particularly friendly, I suspect their real intentions”). The third factor was named “Anger”, since its items refer to the tendency to feel angry without a particular reason (e.g., Sometimes I get very angry for no reason). The last factor was called “Arguing”, since its items refer to the willingness to easily enter into discussions with other people (e.g., “When I do not agree with my friends, I argue with them”).

Analysis of the AVS yielded a three-factor structure containing 12 of the original 14 items which together explained 53.86% of the total variance in responses. The first factor was named “Acceptance of violence against children”, as it contained items such as “A child who is habitually disobedient must be punished physically”. The second factor was named “Acceptance of couple violence”, as it contained items such as “It is a right of the couple to hit the other if they are insulted or ridiculed”. In the original proposal, this factor contained items about violence against children and was named “Family violence”, but in this analysis it only contained items related to violence in couples. The third factor was named “Acceptance of military violence” containing items such as “Our country should be aggressive internationally using military force”.

**Figure 1:**
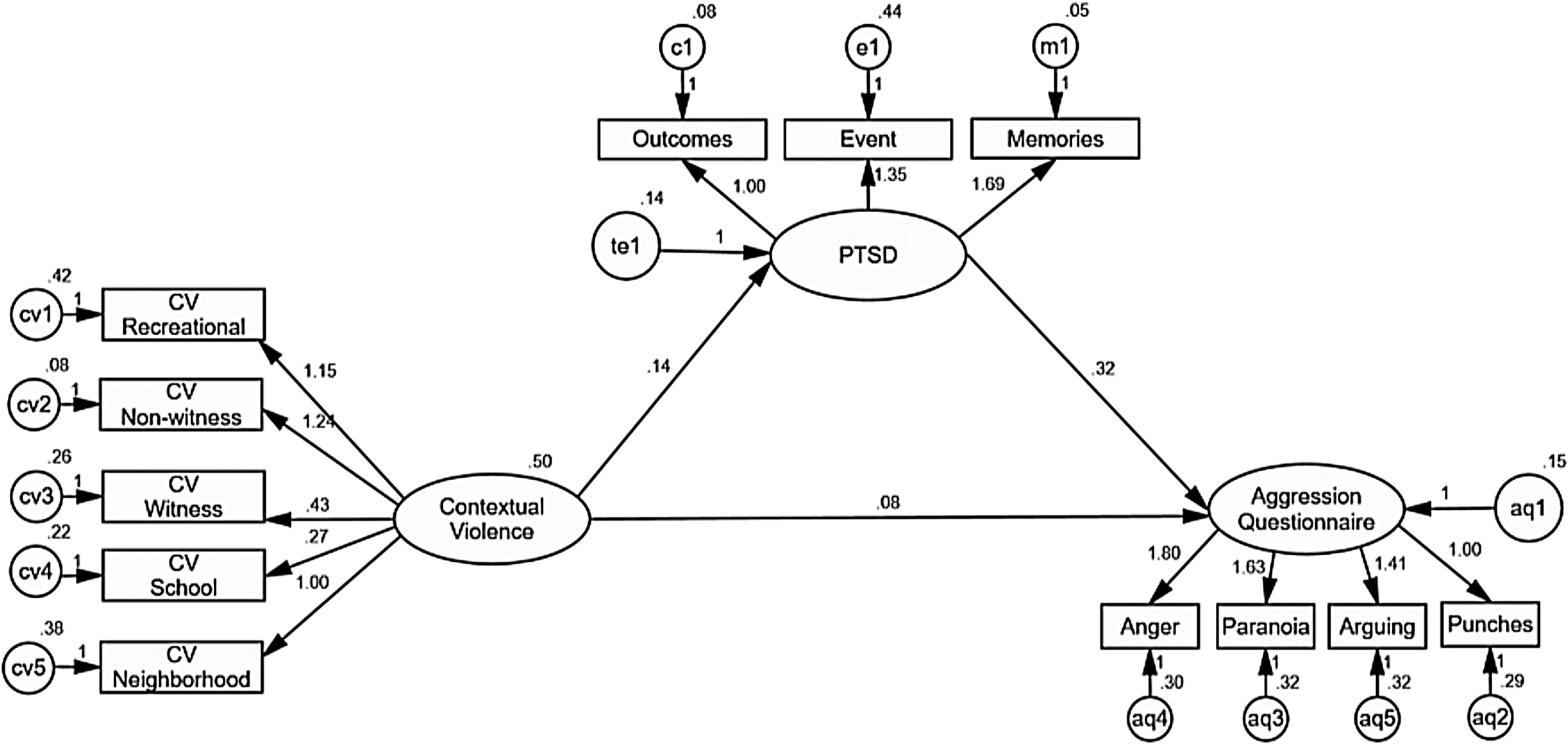
AMOS graphics for a structural equation model analyzing the effects of contextual violence on PTSD, and PTSD on disposition to aggression. Positive and significant predictive effects of contextual violence on PTSD are observed, explaining 6% of the variance. Contextual violence and PTSD also have positive predictive effects on disposition to aggression, explaining 13% of the variance in responses to the Aggression Questionnaire. However, the indicators show a poor goodness of fit for this model (Chi^2^ = 1,404.1851, gl = 512, *p* < 0.001, RMR = 0.03647, GFI = 0.92, AGFI = 88, CFI = 0.943, RMSEA = 0.077 90% CI [0.0623–0.0932]). Unstandardized estimates are shown.

Once the factors were identified, the data were analyzed using the SEM of maximum likelihood in AMOS 22 (Arbuckle, 2013). This initial model, presented in Fig. 1, shows significant factorial loads of all observed variables on their respective latent variables, with CR values among 6.36 and 15.78 with *p* < 0.001., confirming that the structure of the instruments identified by the exploratory factor analysis was adequate for this sample. The model confirms a positive and significant effect of contextual violence on PTSD (*B* = 0.13, CR = 3.88, *p* < 0.001), explaining 6% of its variance. Contextual violence (*B* = 0.08, CR = 2.07, *p* = 0.038) PTSD (*B* = 0.32, CR = 4.25, *p* < 0.001) had positive direct effects on disposition to aggression, which added to an indirect effect of contextual violence on disposition to agresion (*B* = 0.04, *z* = 2.86, *p* = 0.004) (Sobel, 1986), explain together 13% of the variance. However, the fit of this model was not adequate (Chi^2^ = 140.18, gl = 51, *p* < 0.001, RMR = 0.036, GFI = 0.92, AGFI = 88, CFI = 0.94, RMSEA = 0.077 90% CI [0.062–0.092]). An analysis of the residual covariances of the model made it possible to identify that these discrepancies derive mainly from the contextual violence observed variables, which are all part of its latent variable, but only “at school” and “in the neighborhood” variables have a predictive effect on PTSD. This would seem to indicate that the complete construct of contextual violence is not necessary for the prediction of PTSD. The model is more parsimonious if we take only the indicators of contextual violence at school and in the neighborhood, which could also be considered the most common locations for the sample in this study. It is noteworthy that these two factors have a more important effect than variables related to direct exposure to violence such as the “witnessed violence” factor.

**Figure 2:**
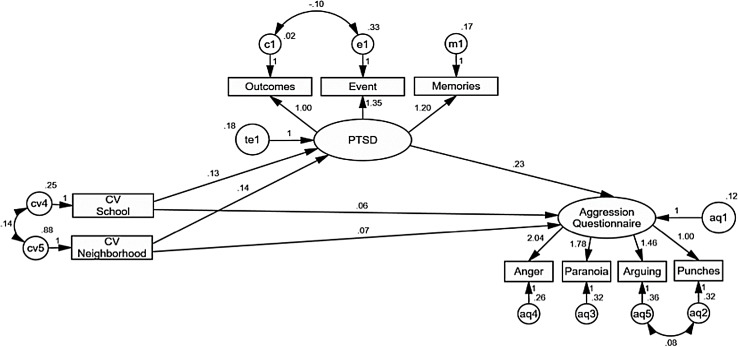
AMOS graphics for a structural equation model analyzing the effects of contextual violence at school and in the neighborhood on PTSD, and PTSD on disposition to aggression. After keeping only contextual violence at school and in the neighborhood, best levels of explained variance were obtained for PTSD (13%) and for aggression (17%), besides best goodness of fit indexes comparing to the previous model (χ^2^ = 41.63, df = 21, *p* = 0.005, RMR = 0.020, GFI = 0.97, AGFI = 0.93, CFI = 0.97, RMSEA = 0.058 90% CI [0.031–0.083]). Unstandardized estimates are shown.

With this model as a backdrop, a new model was tested (see Fig. 2) which preserved only the factors of contextual violence at school and neighborhood for their significant weights on the PTSD. These variables were preserved allowing free covariation between their measurement errors as part of the same construct, but contextual violence was removed as a latent variable, while three of its five original observed variables were eliminated as well. The errors of the AQ variables “punching” and “arguing”, as well as the errors of the variables of the PTSD dimension “event” and “consequences”, were also allowed to freely correlate. It is interesting to note that the covariation between the “event” and “consequences” variables is negative (*B* = −0.10, CR = −3.69, *p* < 0.001), which could indicate a sustained effort to overcome the traumatic event by continuing with everyday life (i.e., those most exposed to traumatic events would be more aware of the importance of continuing their routine activities).

In this new model, contextual violence at school (*B* = 0.13, CR = 2.55, *p* < 0.011) and in the neighborhood (*B* = 0.13, CR = 5.08, *p* < 0.001) together explained the 13% of the variance in responses to the PTSD questionnaire. In turn, PTSD (*B* = 0.23, CR = 3.82, *p* < 0.001), contextual violence at school (*B* = 0.06, CR = 2.53, *p* = 0.011) and in the neighborhood (*B* = 0.06, CR = 1.26, *p* = 0.205) had positive effects on disposition to violence, although this last one is not statistically significant. These direct effects added to indirect effects of contextual violence at the school (*B* = 0.03, *z* = 2.13, *p* = 0.036) and in the neighborhood (*B* = 0.03, *z* = 3.07, *p* = 0.002) (Sobel, 1986), explain 17% of the variance of responses to the AQ. This indicates that 17% of the explained variance of the AQ is given by a direct effect of PTSD, but part of this variance is explained by contextual violence at school and in the neighborhood. This model shows better goodness of fit (RMR = 0.020, GFI = 0.97, AGFI = 0.93, CFI = 0.97), although the Chi-square indicator and RMSEA keep signaling significant discrepancies between the relationships established in the model and those observed in the data (χ^2^ = 41.63, df = 21, *p* = 0.005), and an acceptable possibility to generalize this model to the population from which the sample was extracted (RMSEA = 0.058 90% CI [0.031–0.083]) (Kline, 2011). While Browne & Cudeck (1993) propose that maximum values of 0.08 for RMSEA indicate a reasonable goodness of fit, more demanding criteria such as that of Hu & Bentler (1999) propose that levels above 0.06 indicate that the model could not be generalized to the population from which the sample was extracted.

Considering that women have been especially affected by the violence in Ciudad Juárez, a gender-based comparison of the model was conducted. The indicators show an adequate adjustment of the model without restrictions for CFI and RMSEA (CFI = 0.96, RMSEA = 0.050 90% CI [0.030–0.069]), but not for Chi-squared indicator (χ^2^ = 72.90, df = 42, *p* = 0.002).

**Figure 3:**
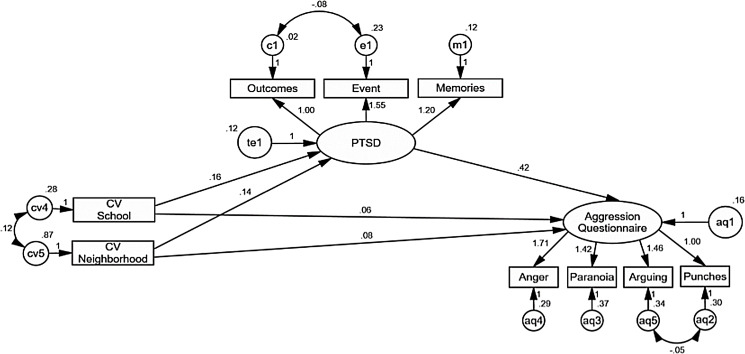
AMOS graphics for a multi-group comparison for women in the sample, analyzing a structural equation model of the effects of contextual violence at school and in the neighborhood on PTSD, and PTSD on the disposition to aggression. Explained variance of PTSD and for aggression decreases notably for men and increases twice for women. Indirect effects of contextual violence at school and in the neighborhood remain statistically significant for women, but not for men. Contextual violence at school remains as a significant predictor of PTSD for women but not for men. Unstandardized estimates are shown.

The prediction effects of the model are significant for the women’s model, with *p* < 0.004 and CR values between 2.92 and 4.43, except for direct effects of contextual violence at school (CR = 0.80, *p* = 0.419) and in the neighborhood (CR = 1.73, *p* = 0.082) on AQ. Indirect effects of violence at school (*B* = 0.06, *z* = 2.18, *p* = 0.028) and in the neighborhood (*B* = 0.06, *z* = 2.65, *p* = 0.008) remain statistically significant, and added to direct effects in the model explain 20% of variance of PTSD and 23% of AQ (Fig. 3). The fact that the significant indirect effects of contextual and neighborhood violence on aggression, and that the direct effects are not significant, point to the PTSD as a mediating variable of the relationship between these forms of contextual violence and aggression. That is, contextual violence in the neighborhood and school increases the predisposition to aggression only when they are high enough to increase PTSD.

**Figure 4:**
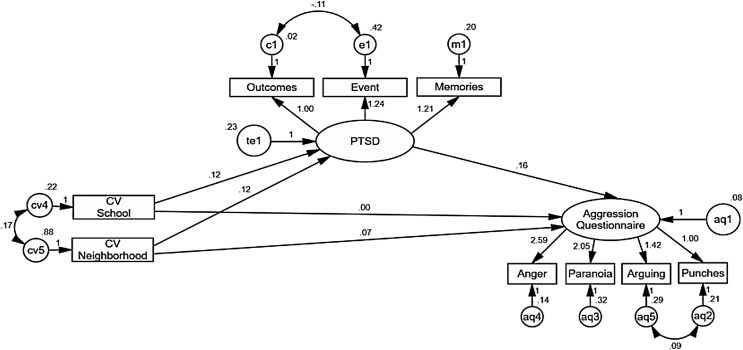
AMOS graphics for a multi-group comparison for men in the sample, analyzing a structural equation model SEM of the effects of contextual violence at school and in the neighborhood on PTSD, and PTSD on the disposition to aggression. Explained variance of PTSD and for aggression decreases notably for men and increases twice for women. Indirect effects of contextual violence at school and in the neighborhood remain statistically significant for women, but not for men. Contextual violence at school remains as a significant predictor of PTSD for women but not for men. Unstandardized estimates are shown.

In contrast, the only statistically significant paths for men’s model are contextual violence in the neighborhood on PTSD (CR = 2.78, *p* = 0.005), PTSD on AQ (CR = 2.59, *p* = 0.010) and contextual violence in the neighborhood on AQ (CR = 2.13, *p* = 0.032). Indirect effects of violence at school (*B* = 0.02, *z* = 1.23, *p* = 0.21) and in the neighborhood (*B* = 0.02, *z* = 1.90, *p* = 0.056) were not statistically significant (Fig. 4). This model results clearly more adequate for women than for men, considering that explained variance decreased to 9% for PTSD and 14% for AQ, almost half than in the women’s model.

These differences between both models would explain the significant discrepancies observed in the χ^2^ indicator of the unrestricted model. As the equivalence between both models was not guaranteed in this first level, no comparison was performed in the following levels of restrictions.

In order to test the hypothesis of the effects of contextual violence and PTSD on the acceptance of violence, the AQ was replaced with the Acceptance of Violence measurement in the previous model. The resulting model did not confirm our hypothesis. There was adequate adjustment of the model (χ^2^ = 13.68, df = 17, *p* = 0.68, RMR = 0.011, GFI = 0.98, AGFI = 0.97, CFI = 1.00, RMSEA < 0.001 90% CI [0.00–0.04]), but an explained variance of 0%.

A possible alternative explanation of the results is that the perception of contextual violence may be affected by participant individual differences, in that more sensitive people would tend to report higher levels of perceived violence. To explore this possibility, high and low groups were formed in the measurement of emotional stability of the TIPI; Gosling, Rentfrow & Swann (2003) based on the median value (4.5). Comparisons were made between both groups using the Student’s *t* test in the indicators of contextual violence at school and in the neighborhood. The results do not identify statistically significant differences in any of the two indicators of violence, with values of *t* < 1.57 and *p* > 0.119 for both comparisons, indicating that reports of contextual violence were not affected by emotional stability.

## Discussion

The current study examined whether degree of contextual violence could determine levels of PTSD and disposition to aggression. The SEM analysis only partially confirmed the hypothesis that contextual violence was predictive of the emotional affectations associated with PTSD. This is because only the indicators of violence at school and in the neighborhood were able to predict any variance in responses to the PTSD instrument. In addition, our analysis showed that these variables were only predictive of PTSD in women, and that the only predictor for men was violence in the neighborhood. As the responses came from a sample of students, it makes sense that the main predictors of PTSD would be violence in the neighborhood and at school, as these are likely to be their most frequented locations. However, the result that violence at school is predictive for women but not men may indicate that school is perceived as a safe space by men only. It is also notable that the factors of contextual violence at school and in the neighborhood, composed of items that mainly relate to hearing that violent acts have occurred, have a greater predictive capacity than factors such as witnessed violence. This may be due to the fact that signs of violence in highly-frequented locations have a greater impact than those in less-frequented areas or in non-specific locations, even if it was personally witnessed.

Indirect effects of contextual violence on AQ were also identified, implying that the PTSD is mediating this relationship. However, the comparison analysis between groups confirmed this result only in the sample of women. This difference may be since the levels of variance explained, both of PTSD and AQ, are markedly higher in women than in men. In this regard, it should be considered that the level of contextual violence in the school reported by women is barely significantly higher than that of men, and their averages of contextual violence in the neighborhood are equivalent. That is, with clearly close levels of contextual violence, the harmful effects on emotional health (PTSD) and its willingness to aggression (AQ) are more important in women. In this regard, it should be recognized that these scales do not evaluate some forms of violence that only women typically suffer such as sexual harassment, whether in work or educational environments, which represents a solid research objective for new projects in this same line.

It would be interesting to explore whether the level of education affected the results. Considering that higher education is associated with rational thinking, it is interesting to consider whether university students who are over-exposed to violence would not accept or justify the use of violence (conscious or rational thinking), even if they tend to rely on violent behaviors (unconscious or reactive acting) in threatening situations. We also propose that community violence studies would benefit from moving beyond common sampling criteria such as age, sex, and education level in order to shed further light on the patterns of social-cognitive functioning among individuals exposed to violence. Due to resource and time restraints, this rational could not be followed in the current study, and as such constitutes a serious limitation. Shahinfar, Kupersmidt & Matza (2001) suggest two ways to accomplish this. Firstly, it is important to carefully consider the modality of exposure to violence of the participants (e.g., if studying effects of indirect exposure, filter those who have had direct exposure to violence). Secondly, the severity of the exposure is a factor that must be emphasized and included in analysis.

The current study confirmed the hypothesis on the role of emotional affectations (PTSD) as potential generators of aggression or at least a tendency to attack others. The direct effects of contextual violence on disposition to aggression show that contextual violence predisposes one to aggression against others in general, both by itself and through its emotional affectations. Several notable differences were identified between men and women models. Although in absolute terms 23% explained variance in the model with women may seem like a small amount, it should be considered that in relative terms, it is twice the amount of variance explained in the model with men. This difference may derive from the increased sense of vulnerability among women from exposure to rape, kidnaping, domestic violence or human trafficking; all these types of crimes with notably high rates in Juárez City.

The elements uncovered in the models presented here could potentially relate to the spread of violence, a phenomenon whose dynamics have been studied among criminal groups in Mexico (Osorio, 2015) but not among general populations. Previous studies have shown that increases in a city’s violence show patterns similar to those of an epidemic (Berkowitz & Macaulay, 1971), where exposure to especially violent events transforms existing norms and the meaning, for example, of “being violent” from an undesirable attribute to a characteristic that provides respect and security. One interpretation of this phenomenon is that as new norms spread among the population to the extent, those endorsing them are more likely to “adjust” (in evolutionary terms). As a consequence, the adopters of these norms can gain access to better resources and develop better solutions for common problems such as exposure to violence (Fagan, Wilkinson & Davies, 2007).

Although no effects on norms related to acceptance of violence were identified in this study, the instruments used did not cover aspects such as acceptance of the possession of weapons, the legitimacy of revenge, the exercise of vigilante justice, or other similar norms which would imply a greater willingness of citizens to join the spiral of violence in their city, that is, what Eisenhammer (2014) has called “bare life”. These elements remain a vein for future research to explore.

## Conclusions

While the model in this investigation is not a definitive model for contextual victimization, we do consider it as a starting point for the study of contextual violence consequences, given that it has been centered mainly on its measure and not on its effects. Future research could also improve by measuring whether the participants have or have not been exposed to direct or indirect violence, in order to limit possible correlates on contextual victimization only. Finally, though this line of research centers on understanding the underlying mechanisms of violence exposure, it must be stressed that the implicit goal of studies in this area should be to improve interventions to reduce aggressive behavior in individuals exposed to violence (Calvete & Orue, 2011), and reduce community violence as a result.

## Supplemental Information

10.7717/peerj.9162/supp-1 Supplemental Information 1 Raw scores for Contextual Victimization by Community Violence (CVCV) scale, Aggression Questionnaire (AQ), Checklist for PTSD Traits Scale, Acceptance of Violence Scale and Ten-Item Personality Inventory (TIPI). Raw scores for each of the administered scales, as well as their sub-scales.

10.7717/peerj.9162/supp-2 Supplemental Information 2 AMOS graphics file for a structural equation model analyzing the effects of contextual violence on post-traumatic stress, and post-traumatic stress on disposition to aggression. Positive and significant predictive effects of contextual violence on post-traumatic stress are observed, explaining 6% of the variance. Post-traumatic stress also has positive predictive effects on disposition to aggression, explaining 12% of the variance in responses to the Aggression Questionnaire. However, the indicators show a poor goodness of fit for this model (Chi^2^ = 144.51, gl = 52, *p* < 0.001, RMR = 0.047, GFI = 0.92, AGFI = 88, CFI = 0.93, RMSEA = 0.077 90% CI [0.063–0.093].

10.7717/peerj.9162/supp-3 Supplemental Information 3 AMOS graphics file for a structural equation model analyzing the effects of contextual violence at school and in the neighborhood on post-traumatic stress, and post-traumatic stress on disposition to aggression. After keeping only contextual violence at school and in the neighborhood, best levels of explained variance were obtained for Post-traumatic stress (13%) and for aggression (14%), besides best goodness of fit indexes comparing to the previous model (χ^2^ = 51.93, df = 23, *p* = 0.001, RMR = 0.036, GFI = 0.96, AGFI = 0.92, CFI = 0.96 and RMSEA = 0.065 90% CI [0.042–0.089]).

10.7717/peerj.9162/supp-4 Supplemental Information 4 AMOS graphics file for a multi-group comparison for men and women in the sample, analyzing a structural equation model SEM of the effects of contextual violence at school and in the neighborhood on post-traumatic stress, and post-traumatic stress on dispo. Explained variance of Post-traumatic stress and for aggression decrease notably for men and increase at double for women. Indirect effects of contextual violence at school and in the neighborhood remain statistically significant for women, but not for men. Contextual violence at school remains as a significant predictor of post-traumatic stress for woman but not for men. Unstandardized estimates are shown.

10.7717/peerj.9162/supp-5 Supplemental Information 5 Codebook for the Aggression Questionnaire, PTSD Checklist traits, Ten-Item Personality Inventory, and Acceptance of Violence Scale.

## Additional Information and Declarations

### Data Availability

The following information was supplied regarding data availability: The raw measurements are available in the Supplemental Files.
